# Subcutaneous Angiomatoid Fibrous Histiocytoma Mimicking Metastatic Melanoma

**DOI:** 10.1155/2012/291623

**Published:** 2012-12-20

**Authors:** E. Sparreboom, C. Wetzels, M. Verdijk, S. Mulder, W. Blokx

**Affiliations:** ^1^Department of Pathology, Radboud University Nijmegen Medical Centre, P.O. Box 9100, 6500 HB Nijmegen, The Netherlands; ^2^Department of Pathology, Maxima Medical Centre, 5500 MB Veldhoven, The Netherlands; ^3^Department of Oncology, Radboud University Nijmegen Medical Centre, P.O. Box 9100, 6500 HB Nijmegen, The Netherlands

## Abstract

Angiomatoid fibrous histiocytoma is an uncommon soft-tissue tumor of intermediate malignancy that is often misdiagnosed initially. As there is not one immunohistochemical marker that consequently stains positive or negative for angiomatoid fibrous histiocytoma, molecular diagnostics are becoming more widely used. So far three translocations have been reported to arise in angiomatoid fibrous histiocytoma: FUS-ATF1, EWSR1-CREB1, or EWSR1-ATF1. We present a case of angiomatoid fibrous histiocytoma on the upper arm of a 40-year-old female, which was initially misdiagnosed as metastatic melanoma in a lymph node. Revision of the pathology revealed an angiomatoid fibrous histiocytoma, which was later confirmed by a EWSR1-CREB1 translocation with molecular diagnostics. Furthermore, we review the relevant literature and provide an overview of all available case reports in the past ten years. This case report illustrates the importance for pathologists of knowing the typical pathology features of AFH and integrating immunohistochemical and molecular findings in order to prevent overdiagnosis of lymph node metastasis of a malignancy.

## 1. Introduction

Angiomatoid fibrous histiocytoma (AFH) is a rare soft-tissue tumor first described by Enzinger in 1979, occurring most commonly in children and young adults [1]. It is classified by the World Health Organization as a fibrohistiocytic tumor of intermediate malignancy [2], with local recurrence rates of 11% and metastatic disease in 1% [3]. AFH develops most frequently on the extremities (65%), followed by the trunk (28%) and head and neck (7%) [4], and is clinically often thought to represent lymphadenopathy, cyst, hemangioma, or Kaposi or Ewing sarcoma [5–7]. Four main histological features usually seen in AFH are a fibrous pseudocapsule, a round or spindle fibrohistiocytic cell proliferation, a pseudoangiomatous pattern, and a plasmalymphocytic infiltrate [5, 8]. The pathological analysis may, however, be difficult as the tumor may mimic lymph node metastasis of another round or spindle cell malignancy, and specific immunohistochemical stains that provide a conclusive diagnosis are lacking [9]. The use of molecular diagnostics can be helpful in establishing a diagnosis in these cases.

We present the case of a 40-year-old woman whose AFH was initially diagnosed as a lesion suspicious for melanoma metastasis. No primary tumor could be discovered after thorough evaluation by other specialists, hence the pathology of the tumor was revised. The final diagnosis was established by means of immunohistochemistry and demonstration of a specific translocation, EWSR1-CREB1 which is described in AFH.

## 2. Case Report

A 40-year-old obese Caucasian woman with no relevant medical history presented at another institution with a fast growing, asymptomatic tumor of the left upper arm that had appeared within several weeks. She was otherwise healthy and had no physical complaints, especially no loss of appetite, weight loss, or night sweats. She had quit smoking 5 years ago and did not use any medication.

On clinical examination she had a normal blood pressure and pulse. There was a small, fixed, and nontender subcutaneous tumor on the left upper arm of approximately 2.0 cm in diameter. Inspection of the rest of the body revealed no other suspicious lesions or lymph nodes. Differential diagnosis of the tumor included lipoma, cyst, venous malformation, or lymph node malignancy upon which the lesion was surgically removed.

**Figure 1:**
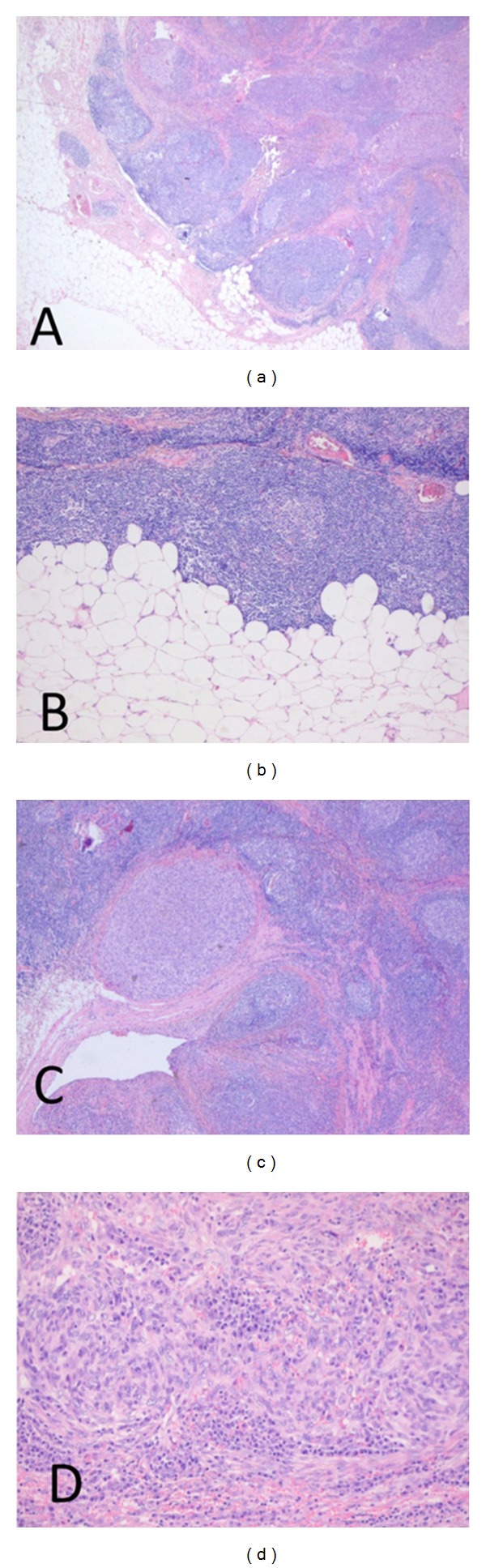
(a) Pathologic review of the tumor showed fibrous septa and nodular texture surrounded by lymphocytic infiltrate. Hematoxylin and eosin stain (12, 5x). (b) Detail image showing the absence of a capsular sinus. Hematoxylin and eosin stain (25x). (c) Image of excised tumor showing fibrous septa and nodular texture surrounded by lymphocytic infiltrate. Hematoxylin and eosin stain (12, 5x). (d) Detail image showing reticular appearance and atypical cytomorphology. Hematoxylin and eosin stain (100x).

**Figure 2:**
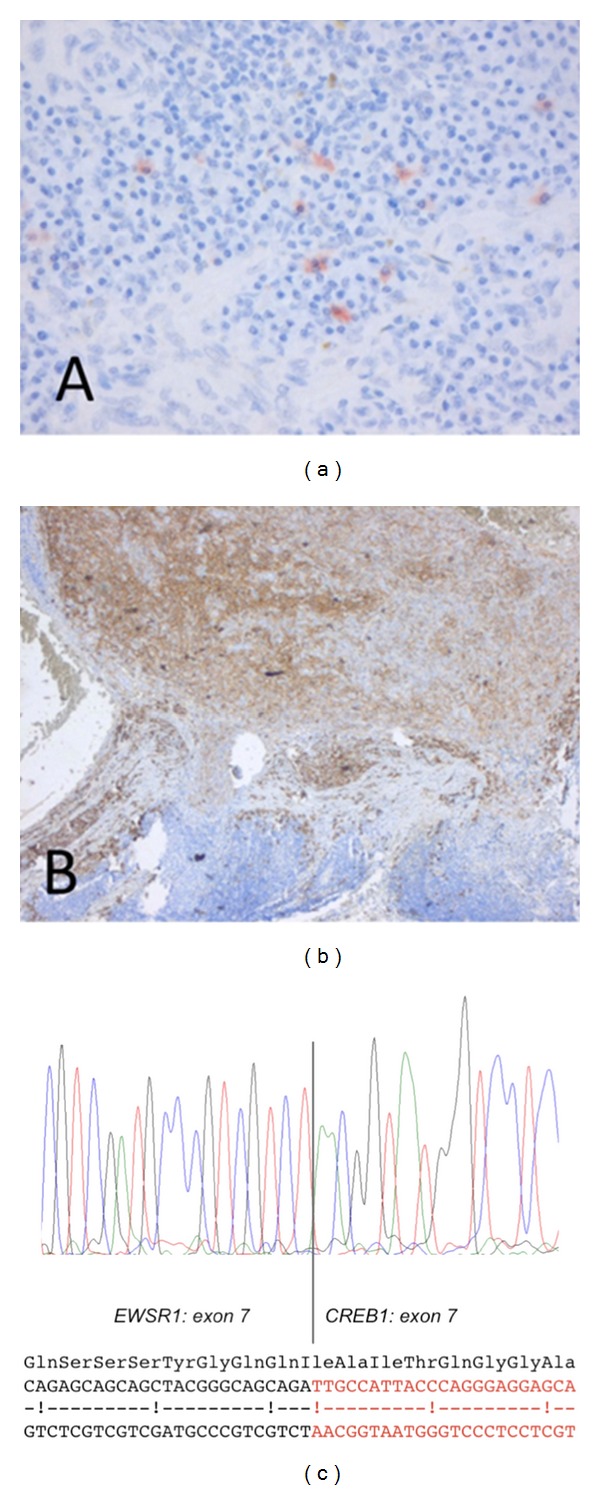
(a) Melan A stain showing focal positivity. Melan A stain (200x). (b) The neoplastic cells were positive for EMA staining. EMA stain (25x). (c) Electrophoretogram of direct sequencing of the junctional region of the EWSR1-CREB1 fusion transcript, with in-frame fusion. The schematic overview shows the EWSR1 nucleotides/amino acids in black and the CREB1 nucleotides in red.

A gray tan nodule measuring 1.2 cm was excised from the subcutaneous fat of the patients' left upper arm. Pathology revealed a radically excised structure resembling a lymph node in which a tumoral process was seen, consisting of a diffuse proliferation of atypical spindle cells. The atypia was specified by undefined cell borders, an amphophilic cytoplasm, large polygonal nuclei and several in part atypical mitoses (Figure 1(d)). Complementary immunohistochemical stains were negative for HMB-45, KL1, cytokeratin AE1/3, and SMa. There was focal positivity for S100 and Melan A (Figure 2(a)) leading to a presumptive diagnosis elsewhere of a melanoma metastasis.

Further clinical investigation of the patient by a dermatologist, ophthalmologist, gynecologist, and surgeon revealed no lesions suspect for the primary tumor. A CT chest and abdomen and PET CT scan also showed no suspect lesions.

Due to uncertainty about the diagnosis and prognosis of the patient, she was referred to the Radboud University Nijmegen Medical Centre (RUNMC) for a second opinion, at which the pathology of the tumor was revised. We confirmed the signs of cellular atypia as described elsewhere, within a spindle cell proliferation with a nodular texture, covered by normal dermis and epidermis without an intraepidermal melanocytic proliferation. The tumor nodules were partially surrounded by fibrous septa and densely vascularized by a capillary network (Figures 1(a) and 1(c)). A dense and extensive lymphatic infiltrate with no evident subcapsular sinus surrounded the spindle cell proliferation (Figure 1(b)).

We considered upon revision the S100 and Melan A staining to be negative in the lesional cells. More immunohistochemical staining, complementary to the previous stains, was performed. The neoplastic cells were positive for CD99 and EMA (Figure 2(b)) and to a lesser extent for CD31. Some capillary vessels stained positive for desmin; however, the tumor cells did not. Other negative stains included CD34, CD30, CD35, Cd10, HHV8, S100, MITF, CD79a, CD20, CD3, CD2, and CD21.

Conclusion of revision of the pathology showed that the tumor, that was initially thought to consist of metastatic melanoma within a lymph node, was in fact a different type of spindle cell tumor surrounded by an extensive lymphatic infiltrate and densely vascularized by capillaries and a few larger vessels. Given the age of the patient and location of the lesion combined with the histopathologic findings, the diagnosis was adjusted into angiomatoid fibrous histiocytoma. Additional molecular research, performed in support of this diagnosis, showed a gene fusion mutation of EWSR1-CREB1 t(2;22)(q33;q12) (Figure 2(c)), while mutations in BRAF and NRAS genes, common in cutaneous melanoma, could not be detected.

## 3. Discussion

Angiomatoid fibrous histiocytoma is a mesenchymal tumor of intermediate malignancy of unknown differentiation. Although it was originally reported to arise most frequently on the extremities of children and young adults, many case reports found in the literature describe AFHs developing on other sites of the body, such as the mediastinum [4], bone [10], intrapulmonal [11], or intracranial [11]. A complete overview of sites of origin of AFH in available case reports published in the past 10 years can be found in Table 1. In total 18 case reports on AFH were found in the past decade. In addition some case series were reported [5, 9].

Table 1 shows that most AFHs are found in patients in the first 3 decades of life, though patients aged 80 or more with AFH have also been reported [4, 7]. AFH on the upper extremity of a healthy middle-aged woman can therefore be considered a classic presentation of this tumor.

Due to the fact that AFHs are rare, the typical histological features are relatively unknown to pathologists which can lead to an erroneous diagnosis of malignant disease (Table 1). This is most likely due to the typical dense lymphocytic infiltrate surrounding the tumor, suggestive of a tumor metastasis to a lymph node. In one other reported case (case 1, Table 1), as in our case, metastatic melanoma was a differential diagnostic consideration.

In our case the initial diagnosis was metastatic melanoma in a lymph node, due to the dense capsule and the surrounding plasmalymphocytic infiltrate and focal aspecific staining of lesional cells for melanocytic markers [12, 26, 27]. Careful revision eventually revealed the absence of structures normally found in lymph nodes, such as a subcapsular sinus and triggered further research on the origin of the present tumor.

Interpreting immunohistochemical staining results can be complex, as there is not one immunohistochemical marker that consequently stains positive for AFH. As indicated in Table 1, the majority of studies have reported AFHs to stain positive for CD68 [8, 28], desmin, EMA, and vimentin. Within a small percentage of AFHs other muscle markers such as HHF-35 and calponin also stain positive [29]. The AFH in the present case report showed positive staining for CD99 and EMA, though desmin seemed positive only in the surrounding capillary epithelium and CD68 in the intralesional dendrites. Double immunoreactivity for EMA and desmin is reported to be a diagnostic clue for AFH [5].

Nevertheless, immunohistochemistry has a limited role in establishing the diagnosis of AFH. The knowledge of the existence of this rare entity with its typical pathology features is therefore vital in preventing misdiagnosis.

As the molecular genetics of AFH become increasingly understood, genetic testing is utilized more widely to support the diagnosis of this entity. There are three translocations resulting in fusion genes associated with AFH: FUS/ATF (t(12;16)(q13;p11)) [30], EWSR1/ATF1 (t(12;22)(q13;q12)) [23], and EWSR1/CREB1 (t(2;22)(q33;q12)) fusion genes [31]. In available case reports published in the past 10 years, the EWSR1/ATF1 (t(12;22)(q13;q12) fusion was most commonly found (Table 1). In a series of 9 AFH Antonescu et al. reported that EWSR1-CREB1 was the predominant gene fusion in AFH present in 8/9 cases [31]. In our case, the patient was also tested positive for the EWSR1/CREB1 (t(2;22)(q33;q12)) fusion gene, hereby supporting the diagnosis of AFH. The EWSR1-CREB1 translocation is not unique to AFH but also present in clear cell sarcoma of the gastrointestinal tract and soft tissue [32].

In conclusion, AFH is a rare disease that is often misdiagnosed initially. Prognosis is generally good following wide surgical excision, with low potential of local recurrence and metastasis.

Our case report illustrates the importance for pathologists of knowing the typical pathology features of AFH and integrating immunohistochemical and molecular findings in order to prevent overdiagnosis of lymph node metastasis of a malignancy.

One year following excision, our patient is well without signs of local recurrence or metastasis.

## Figures and Tables

**Table 1:** Overview of all case reports on angiomatoid fibrous histiocytoma between 2002 and 2012.

Article	Patient age	Localization	Differential diagnosis	Immunohistochemical staining	Molecular diagnostics
(1) Mansfield et al., 2010 [12]	M, 25	Left axilla	Metastatic melanoma, rhabdomyosarcoma	*Positive:* vimentin, EMA, and CD68 *Negative*: S100, Melan A, HMB45, myogenin, desmin, smooth muscle actin, and keratin	—
(2) Song et al., 2011 [13]	F, 23	Palatum	Necrotizing sialometaplasia, tertiary syphilis, and Wegener's granulomatosis	*Positive:* proliferating cell nuclear antigen, S-100, CD31, and desmin *Negative:α*-smooth muscle actin, p53, survivin, CD68, and TNF-*α*	—
(3) Moura et al., 2011 [4]	M, 80	Mediastinum	—	*Positive:* EMA and desmin *Negative:* S100, Melan A, smooth muscle actin, anticytokeratin CAM 5.2, anticytokeratin OSCAR, chromogranin, synaptophysin, myogenin, and glial fibrillary acidic protein	EWSR1/CREB1 fusion
(4) Ajlan et al., 2010 [14]	F, 28 F, 85	Left shoulder Right upper arm	—	—	—
(5) Cernik et al., 2009 [15]	M, 6	Left forearm	Hemangioma	*Negative:* CD34, reticulin, and pancytokeratin	—
(6) Mangham et al., 2010 [10]	M, 11	Right upper arm	—	*Positive:* desmin, EMA, and cytokeratin (AE1/AE3) *Negative:* CD31, CD34, CD45, S100, smooth muscle actin, HMB45, myoD1, myoglobin, myf4, and myogenin	EWSR1-ATF1 fusion
(7) Ochalski et al., 2010 [11]	M, 35	Intracranial	Meningioma	*Positive:* vimentin, desmin, and CD68 *Negative:* MYOD1, myoglobin, and myogenin	Rearranged ESWR1 gene
(8) Ren et al., 2009 [16]	M, 46	Intrapulmonal	—	*Positive:* EMA, desmin, CD163, and CD68 *Negative:* S100 protein, smooth muscle actin, CD34, cytokeratin, anaplastic lymphoma kinase-1, CD21, CD23, and immunoglobulin light chains	EWS/ATF1 gene fusion
(9) Weinreb et al., 2008 [17]	M, 8	Scalp	Pleomorphic fibrous histiocytoma	*Positive:* CD99, Factor 3a, desmin, and SMA *Negative:* S100, pankeratin	EWSR1 translocation on 22q12
(10) Martelli et al., 2008 [6]	F, 7 (HIV+)	Right knee	Benign epithelial cyst, mesenchymal tumor	*Positive:* desmin *Negative:* S100, CD68, and smooth muscle actin	—
(11) Dunham et al., 2008 [18]	M, 25	Intracranial	—	*Positive:* v imentin, CD68, S100, CD31, and desmin *Negative:* CD34, glial fibrillary acidic protein, neurofilament, synaptophysin, CAM 5.2, HMB-45, and progesterone receptor stains	EWS/ATF-1 gene fusion
(12) Koletsa et al., 2007 [19]	F, 28	Left leg	—	*Positive:* vimentin, CD68 *Negative:* SMA, desmin, CD34, CD31, factor VIII-related antigen, S-100, EMA, and cytokeratins cam5.2, AE1/AE3 and 17	—
(13) Hallor et al., 2007 [20]	M, 11 M, 10	Paravertebral Right clavicular region	—	—	EWSR1-ATF1 fusion genes (2x)
(14) Pratibha and Ahmed, 2006 [21]	F, 13	Left lower jaw	—	*Positive:* CD68 *Negative:* desmin and keratin	—
(15) Lai et al., 2006 [22]	F, 28	Neck	—	—	—
(16) Hallor et al., 2005 [23]	M, 9	Right elbow	Reticulum cell tumor, Rosai-Dorfman disease	*Positive:* CD68, vimentin *Negative:* S100, CD1a, cytokeratin, EMA, CD21, CD31, CD34, HMB45, and Melan A	EWSR1-ATF1 fusion gene
(17) Hothi et al., 2004 [24]	M, 13	Left thigh	—	—	—
(18) Raddaoui et al., 2002 [25]	M, 38	Right upper arm	High-grade sarcoma	*Positive:* vimentin, calponin, CD99, and desmin *Negative:* keratin AE-1/AE-3, S100, CD34, CD57, macrophage specific marker, MSA, and SMA	FUS/ATF1 fusion gene
